# Climate change and mental health in India: a narrative review of vulnerabilities, impacts, and resilience pathways

**DOI:** 10.3389/fpubh.2025.1686876

**Published:** 2025-11-28

**Authors:** Banani Basistha, Fayaz Ahmad Paul, Kangkan Bhuyan, J. S. R. Prasad, Arif Ali

**Affiliations:** 1 Amity Institute of Psychology and Allied Sciences (AIPS), Amity University, Noida, Uttar Pradesh, India; 2 Department of Social Work, Rajagiri College of Social Sciences, Kalamassery, Cochin, Kerala, India; 3 Department of English, Chatia College, Assam, India; 4 School of Humanities, University of Hyderabad, Hyderabad, India; 5 Department of Psychiatric Social Work, Institute of Human Behaviour and Allied Sciences (IHBAS), New Delhi, India

**Keywords:** climate change, mental health, India, vulnerable populations, extreme weather, community interventions

## Abstract

India’s rapid urbanization, population growth, and reliance on climate-sensitive sectors make it highly vulnerable to the mental health impacts of climate change. Extreme weather events and environmental degradation disproportionately affect vulnerable populations, yet mental health consequences remain under-addressed in policies and interventions. This narrative review examines the effects of climate change on mental health in India, highlighting risks for vulnerable groups and underscoring the need for climate-sensitive mental health policies and interventions. A comprehensive literature search was conducted using PubMed, Scopus, Google Scholar, and Web of Science. Searches covered the period 2000–2024, and included articles reported in English; we included empirical studies, reviews, case reports and government documents focused on Indian populations and excluded non-India studies and papers without mental-health outcomes. Study quality was appraised using standard checklists, and data were synthesized thematically to identify population-specific vulnerabilities and psychosocial outcomes. Climate change exacerbates anxiety, depression, PTSD, and stress among children, women, the older adults, and rural and urban communities. Mental health must be integrated into India’s climate adaptation and disaster management strategies. Strengthening community-based interventions, awareness programs, and mental health infrastructure will enhance resilience against climate-induced psychological distress. The review applies an eco-social framework to conceptualize pathways linking climate stressors, displacement, and socio-economic disruption to mental health outcomes and identifies the need for longitudinal, culturally validated, and implementation-oriented research.

## Introduction

India faces significant environmental challenges, with climate change being a critical concern. As the world’s most populous country, the strain on resources, rising energy demands, and environmental degradation have intensified. Its geography, diverse climate, and dense population make it highly vulnerable to climate change, evident in the increasing frequency of extreme weather events. The recent Wayanad landslides in Kerala on July 30, 2024, caused by an extraordinary rainfall event, led to over 200 deaths and severe property damage. A rapid analysis by the World Weather Attribution found that climate change has increased the likelihood of such extreme rainfall events by 10% (1). This event has been linked to intensified extreme rainfall attributable to climate change (1) and subsequent studies have reported increased PTSD and depression in directly affected communities (2, 3). Climate change also deeply affects mental health, particularly among at-risk groups such as children, women, the older adults, and both rural and urban communities. This paper is an attempt to highlight how various stress related disorders, PTSD, anxiety and depression are brought forth by weather events of extreme nature such as cyclones, floods, droughts and heatwaves. Emphasis is also given on the specific hardships faced by old people and expecting mothers along with what separates the rural experiences from the urban ones.

Interconnected problems of mental health and climate change need to be addressed immediately in countries like India where the at-risk sections of the population are severely affected by changes in the environment and rising extremities of weather conditions. Policy and climate change frameworks still do not give the much-needed importance to mental health despite having adequate knowledge of the effects of climate changes. The objective of this review was to consolidate findings from empirical studies, case reports, policy documents in order to analyze how various climate change factors affected the mental wellbeing of the people primarily based on agriculture, urban–rural populations and those in the coastal regions of India. This review also emphasized on the vulnerable sections of the society comprised of the older adults, women, and children. Analyses were made of various psychological effects and differences in demography by way of which the present research makes the case for understanding the need for incorporating treatments for mental health that are resilient to climate. This comprehensive analysis underscores the importance of integrating mental health into climate change discourse, fostering targeted strategies to enhance community resilience and wellbeing.

In this review, the term *mental health* refers broadly to emotional, cognitive, and social wellbeing encompassing a person’s ability to cope with stress, maintain relationships, and function productively (40). *Psychological distress* denotes a spectrum of emotional suffering, including anxiety, sadness, fear, or hopelessness, which may not necessarily meet diagnostic criteria for a disorder but reflect impaired wellbeing during or after climate-related stressors. *Psychiatric disorders* are used specifically for clinically diagnosed conditions such as depression, post-traumatic stress disorder (PTSD), and generalized anxiety disorder as reported in empirical studies. Emotional challenges *or* psychosocial impacts describe adaptive or situational emotional responses to environmental adversity. These distinctions are applied consistently throughout the review to enhance conceptual clarity. Using the eco-social framework, women’s vulnerability to climate-related mental health impacts can be understood through the intersection of gendered social roles, economic dependence, and limited adaptive capacity. Within this framework, the mental health impacts of climate change among children, women, and older adults are understood not as isolated outcomes but as interlinked processes shaped by socioeconomic, geographic, and institutional inequities.

## Conceptual model of climate change and mental health in India

The relationship between climate stressors and mental health outcomes in India can be understood as a multi-level causal process involving environmental, social, and psychological mediators. Extreme weather events such as heatwaves, floods, cyclones, and droughts (environmental stressors) disrupt livelihood security, displacement, and community stability (socio-economic disruptions). These, in turn, generate chronic stress, loss, and trauma, leading to adverse mental health outcomes such as anxiety, depression, PTSD, and suicide. Moderating factors include individual resilience, social capital, access to health care, and cultural coping mechanisms, which can either buffer or amplify the impact.

## Materials and methods

### Methodological approach

This narrative review was conducted in accordance with the Scale for the Assessment of Narrative Review Articles (SANRA) criteria to ensure methodological transparency, scholarly rigor, and conceptual clarity (88). A narrative review design was adopted as it allows for the integration of multidisciplinary evidence drawn from environmental sciences, psychiatry, psychology, sociology, and public health domains essential to understanding the multifaceted relationship between climate change and mental health in the Indian context. Unlike systematic reviews that depend on standardized methodologies and homogeneous datasets, the narrative approach enables conceptual synthesis and contextual interpretation of diverse empirical and theoretical sources. This flexibility is particularly appropriate for an emerging area like climate change and mental health, where evidence spans ecological, psychosocial, and policy dimensions.

### Literature search strategy and selection criteria

We searched PubMed, Scopus, Web of Science and Google Scholar from 2000 to 2024 using combinations of keywords and MeSH terms including “climate change,” “*heatwave,” “flood,” “cyclone,” “mental health,” “PTSD,” “depression,” and “India.”* Inclusion criteria were: (1) empirical studies, reviews, case reports, or government documents that reported mental-health outcomes associated with climate or weather events in Indian populations; (2) outcomes including PTSD, anxiety, depression, suicide or other psychosocial impacts; (3) studies reporting original data or national/regional surveillance. Exclusion criteria were studies not reporting mental-health outcomes, modeling papers without mental-health data, non-Indian populations, and commentaries without empirical data. Articles were screened through titles, abstracts, and full-text reviews to ensure relevance and quality; full texts were retrieved for potentially relevant studies and decisions on inclusion were made by consensus. We extracted study design, sample, exposure type, outcome measures, and key findings into a standardized data sheet.

### Study designs and strength of evidence

The reviewed literature included 23 cross-sectional studies, 5 cohort studies, 6 qualitative or mixed-methods studies, and several governmental or policy documents. Most studies were cross-sectional, limiting causal inference, while few used longitudinal follow-up to track psychological sequelae of climate events. Stronger evidence emerged from population-based studies in Kerala, Odisha, and Assam employing validated tools such as the GHQ, PHQ-9, and PCL-5. However, variability in methodology and outcome measures restricts comparability across studies. Future research in India should emphasize longitudinal and intersectoral designs integrating objective mental health assessments with climate and livelihood indicators.

### Quantitative evidence and prevalence patterns

Although many studies qualitatively describe psychological distress following climate events, few report standardized prevalence estimates using validated scales. Studies using the General Health Questionnaire (GHQ), the Patient Health Questionnaire (PHQ-9), and the Post-Traumatic Stress Disorder Checklist (PCL-5) show PTSD prevalence ranging from 15 to 39% among flood and cyclone survivors, and depression rates between 20 and 45% depending on exposure severity (2–4). However, most studies are cross-sectional, limiting causal inference, and national prevalence data specific to climate-related psychological morbidity remain unavailable. Incorporating standardized quantitative measures would enhance comparability across studies and improve understanding of population-level burden.

### Quality appraisal

To assess credibility, quantitative observational studies were appraised using the Newcastle–Ottawa Scale for cohort and case–control or an adapted checklist for cross-sectional studies; qualitative and mixed-methods studies were appraised using the CASP/JBI critical appraisal tools. Government reports and case reports were judged against transparency and data-source criteria. Appraisal results were used to weight interpretation in the narrative synthesis.

### Climate change: a growing concern for India, impacts and urgent actions

India’s changing climate stems from land use changes, human activities like black carbon and greenhouse gas emissions, and natural factors such as solar variations (5–7). With its far-reaching effects on the environment, society, and economy, climate change is a major global issue. Ghosh (8) defines it as long-term shifts in climate patterns, marked by changes in mean and variability lasting decades or more. India’s rapid population growth is driving higher energy demand for residential, industrial, and infrastructural needs. The nation’s fast-growing economy relies heavily on fossil fuels like coal, oil, and natural gas, making it a top contributor to global greenhouse gas emissions. Urban expansion and a rising middle class have further increased energy consumption, intensifying carbon dioxide and pollutant emissions. The transportation sector also plays a major role, with growing vehicle ownership and reliance on personal transport significantly boosting carbon dioxide, nitrogen oxides, and particulate matter emissions. Agriculture is crucial to India’s economy but significantly contributes to climate change, particularly through methane emissions. Rice cultivation, widespread in India, generates methane due to the anaerobic conditions in flooded fields. Livestock farming, especially cattle, adds to emissions through enteric fermentation. The use of synthetic fertilizers releases nitrous oxide, a potent greenhouse gas. Deforestation and land-use changes further exacerbate climate change, as forests help absorb carbon dioxide. Expanding agriculture, infrastructure, and urbanization have reduced forest cover, with India losing 414,000 hectares of humid primary forest from 2002 to 2023, accounting for 18% of total tree cover loss.

Since 2001, India has lost 2.33 million hectares of tree cover, leading to 1.20 billion tons of CO₂ emissions. Rapid urbanization is reshaping India as people migrate for better opportunities, driving infrastructure expansion and reducing green spaces. This shift disrupts ecosystems and amplifies the urban heat island effect, raising temperatures. In rural areas, biomass burning for cooking and heating emits black carbon, worsening global warming and air pollution. Crop residue burning in Punjab and Haryana further contributes to pollution. Climate change is already disrupting India’s environment. Growing seasons are changing (9), affecting productivity and livelihoods, with nearly 50% of Indians working in climate-sensitive sectors (10). The erratic southwest monsoon and extreme weather events like heatwaves, droughts, and cyclones are reducing agricultural output (11). Fossil fuel combustion has intensified heatwaves, making them 1.5 °C hotter and 2.8 times more frequent. India’s temperature rose by 0.7 °C from 1901 to 2018, with 2024 recording highs above 52 °C (12). Rising sea levels, increasing from 1.9 mm per year (1971–2006) to 3.7 mm (2006–2018) (13), threaten coastal populations. Without action, the consequences will be severe.

### Geographical and climatic diversity in India

India’s tropical monsoon climate is shaped by its diverse geography, leading to significant regional and seasonal variations. The Himalayas act as a barrier, preventing cold Central Asian winds from reaching northern India, keeping winters milder than other regions at similar latitudes. Summers in the north are dry and hot, resembling equatorial climates. While most of India experiences distinct wet and dry seasons, regions like Ladakh and the Thar Desert receive little to no rainfall due to their unique geography. The northeast remains hot and humid, whereas the northwest is dry and arid (14). Rainfall varies widely, with Jaisalmer receiving minimal precipitation and Meghalaya experiencing heavy rainfall, largely due to the monsoon winds, which are crucial for agriculture. Over 80% of India’s annual rainfall comes from the southwest monsoon, lasting from June to October. The southwest, with its moist tropical climate, receives over 1,500 mm of rain annually, while the northeast monsoon brings less rainfall, mainly affecting the southeast. Coastal areas benefit from stable temperatures and consistent rainfall, whereas inland regions experience extreme temperature shifts. The Himalayas have the greatest annual temperature variations due to altitude, while the Thar Desert sees high diurnal temperature swings. India’s latitudinal stretch from 8°N to 37°N results in tropical climates in the south and harsh winters in the north (15). Global phenomena like El Niño and La Niña significantly impact India’s climate by disrupting monsoon patterns, causing droughts or floods that directly affect agriculture and millions of livelihoods.

### Why the climate change discourse in India needs to address mental health

**Figure 1:**
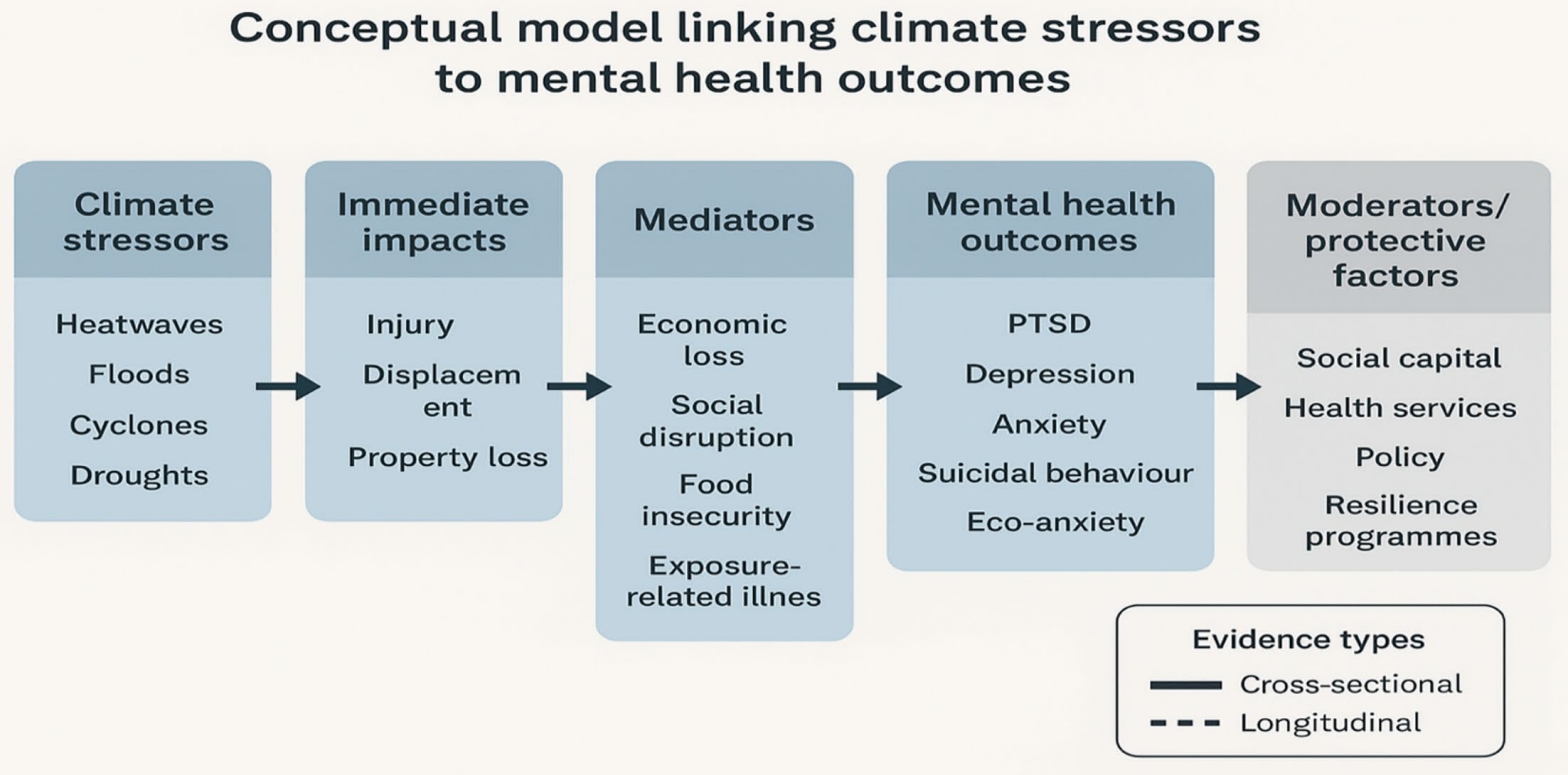
Conceptual model of climate change and mental health pathways in India.

India faces mounting climate change challenges due to its diverse climate and socio-economic inequalities. Extreme weather events like floods, heatwaves, droughts, and cyclones, along with rising temperatures and shifting rainfall patterns, pose serious risks, particularly for marginalized communities, rural populations, indigenous groups, and vulnerable demographics such as children, women, and the older adults. Considering India’s reliance on agriculture and coastal economies, climate change significantly impacts mental health. Due to failures in crop production which results in reduced income and reduced security of food, the farmers who were already facing financial hardships now suffer more psychologically. Data from the National Crime Records Bureau (NCRB) showed a significant growing concern regarding the agrarian sector. Their report from the year 2023 documented a total of 10,786 suicides among farmers and agricultural workers (16, 17). The highest number of these deaths were from Maharashtra, which was 38.5% of the total. Karnataka followed, reporting 22.5% of the farmer suicides (16, 17). The total figure of 10,786 suicides from the farming sector included 4,690 farmers and 6,096 agricultural workers (16, 17). These incidents made up 6.3% of the total suicides recorded across the nation during that year, which stood at 171,418. Other than Maharashtra and Karnataka, Andhra Pradesh (8.6%), Madhya Pradesh (7.2%), and Tamil Nadu (5.9%) registered the next highest numbers of suicides objective (16, 17). People living in the coastal regions mostly dependent on fishing become threatened due to severe cyclones and the rise in sea levels which results in loss of homes and livelihoods. They become uncertain about what the future holds for them given that their lives are governed mostly by the changes in the climate. Surprisingly in Odisha, it was noticed that climate change has highly impacted the ways of life and societal resilience of the fishing community. Gradual intensification of climatic vulnerabilities and extreme weather conditions have interrupted fishing activities which resulted in decrease of their income security (18). Researchers and policy makers in India mostly ignore how climate changes affects mental health of the people despite the spread of awareness on this topic. Strategies of disaster management seldom focuses on psychological after-effects thereby resulting in the heightened importance of looking at factors impacting mental health risks (Figure 1).

### Mental health risks of climate change: vulnerable populations in India

Weather events which are extreme in nature, changing conditions of the environment, and socioeconomic consequences can be considered to be some of the ways by which climate change affects the mental health of people. Prior to these events and also after them, it is possible that mental health may be affected (19). In countries like India, Brazil and China, impacted communities consisting of refugees, minorities and homeless people are found to be specifically impacted (20, 21). The term vulnerable populations in the contemporary context of India refers to specific groups of individuals who were considered more prone to suffering adverse mental health consequences directly attributable to climate change impacts which included socioeconomic, demographic, geographic, and clinical factors collectively shaping a person’s ability to anticipate and recover from negative effects. The term socioeconomic vulnerability has been used in the context of those populations whose survival depended on the specific sectors sensitive to climate shifts such as agriculture and fishing- a dependence rendered them more vulnerable to risks of climate change and the resulting mental health issues. Demographic vulnerability included traits such as age and gender and it may be mentioned that children, women, and the older adults often experienced greater exposure to environmental stressors and had therefore their resilience to adapt to changing situations gradually decreased. Similarly, the term geographic vulnerability concerned groups living in high-risk or specific locations involving coastal, drought-prone, or flood-prone areas, where environmental hazards posed a direct threat to both their secure livelihoods and mental wellbeing.

In addition to these vulnerabilities, Compton and Shim (22) stated that changes in climate is an important determinant of the society related to mental health which impacts those who are more likely to have experiences of psychiatric issues. The present study defined clinical vulnerability as encompassing those individuals who had pre-existing mental or physical health issues- persons who faced an increased risk of experiencing psychological distress and trauma following climate-related events such as people with existing anxiety disorders or depression often saw their symptoms worsen during extended periods of heatwaves, floods, or droughts. This occurred because of increased stress and disruption to their normal daily lives. Individuals who already lived with post-traumatic stress disorder (PTSD) could relive past traumatic events or develop new distressing memories when they witnessed or endured natural disasters like cyclones or landslides (23, 24). Heat stress due to high ambient heat temperature may be responsible for 60,000 additional suicides in India (25, 26). It is important to note that farmers have high rates of anxiety, emotional distress, depression, and suicide risk (27). Mental health conditions can develop faster or may even worsen in those people with lower socio-economic status or no social support in the periods following traumatic events (20, 28).

### Extreme weather and mental health in vulnerable Indian communities

Prolonged periods of very high temperatures, or heatwaves, are associated with health problems and increased mortality rates. PTSD, anxiety, mood disorders, violence, substance misuse, and also a greater risk of suicide have all been linked to frequent exposure to such events (20, 29). Cyclones, with their life-threatening winds, rains, and flooding, lead to trauma, property loss, and displacement, often causing PTSD, anxiety, and emotional detachment, making recovery difficult. The immediate aftermath of a cyclone involves powerful winds, heavy rainfall, and flooding thereby creating trauma stemming from life-threatening situations, property loss, and displacement. This had resulted in psychological difficulties for the affected persons including post-traumatic stress disorder (PTSD), which manifested as flashbacks, nightmares, severe anxiety, and emotional detachment. These are the very symptoms which made personal adjustment following the disaster more difficult (3). The super-cyclone that devastated 12 coastal districts of Orissa in October 1999 was an event that caused over 20,000 deaths and widespread property destruction and later research subsequently correlated it with a high occurrence of mental health conditions among the survivors, and after that anxiety, sadness, and PTSD were frequently observed, particularly among vulnerable groups which included children, the older adults, people from lower socioeconomic strata, and those who had suffered significant life trauma. Increased suicidal thoughts followed these mental health issues (3).

Floods are increasing in frequency and intensity, creating widespread psychological distress. In Assam (in 2022), young adults impacted by floods experienced higher distress, lower health satisfaction, and diminished quality of life compared to unaffected individuals (4). Relocation due to flooding, with its financial and emotional toll, further increases stress. In Kerala (in 2018), PTSD, anxiety, and depression persisted for over a year, with women experiencing higher distress levels (2). Droughts, particularly in farming areas, cause water shortages and economic difficulties, leading to increased stress, anxiety, and depression, and are linked to higher suicide rates among farmers (29, 30). Beyond immediate weather events, climate-induced environmental changes disrupt daily life and resources, further intensifying mental health challenges. A clear distinction regarding psychological fallout existed between sudden calamities and ongoing climate pressures when compared with other climate stressors, acute disasters, such as floods and cyclones. This brought on immediate reactions mainly including PTSD, intense fear, and acute anxiety disorders which arose from the abrupt encounters with threat and substantial loss (2–4). Long-term environmental factors like droughts and extended heatwaves caused mental health to deteriorate slowly and it was most commonly observed as depression, a sense of hopelessness, and suicidal behaviour (29, 30). Services that focuses on crisis related matters are crucial for survivors of acute disasters and various communities undergoing chronic environmental stress, however, needed long-term psychosocial help and programs to build resilience.

### Impact of climate change on children’s mental health and academic performance in India

Climate change threatens both the physical and mental health of children, with serious consequences. Increased awareness of climate dangers often causes anxiety and concern, particularly in India, where children study environmental education to raise awareness of global challenges. However, learning about ecosystem degradation and loss of biodiversity can lead to anxiety, helplessness, and despair (31). Climate change also worsens food security through droughts, floods, and unpredictable monsoons, decreasing agricultural productivity and worsening malnutrition and stunted growth, especially in rural children. These impacts increase developmental and emotional vulnerabilities, with children more likely to experience PTSD and depression than adults (20, 32). Kar et al. (33) studied children affected by a super-cyclone in Odisha, India, finding many had PTSD. PTSD was more severe in highly affected areas, and anxiety about future climate events intensified emotional distress. In a study by Mathew et al. (34), half of students from Kerala schools still struggled with distressing memories after a flood, indicating a high rate of probable PTSD. Climate-induced displacement disrupts social ties, worsening depression, anxiety, and emotional challenges, such as nightmares and social withdrawal. Children may struggle with concentration, motivation, and academic performance due to these emotional impacts (34).

Rising temperatures and changing weather patterns have increased the spread of vector-borne diseases like malaria and dengue, weakening children’s immune systems. Malaspina et al. (35) found that prenatal exposure to extreme temperatures, especially with other stressors, can impair brain function and behaviour, leading to cognitive issues, social dysfunction, and poor educational outcomes, even without psychiatric disorders. Extreme weather events often result in school closures, disrupting education and social connections. Climate-driven migration further complicates educational stability, as children must adapt to new schools and environments, affecting their learning and mental health. While research on PTSD and academic performance in India is limited, Mathew et al. (34) found that 45% of students with PTSD after floods had lower academic performance, which hindered their cognitive and social development and threatened their long-term prospects. Among adolescents and children, conditions of anxiety related to the climate has started gaining distinct recognition as a psychological phenomenon. Various associations have been discovered between the outcomes of children’s mental health and anxiety related to changes in climate according to a recent study in Pakistan which prioritizes looking at eco-anxiety within healthcare and educational systems as an urgency (36).

Worrying about the climate is not just circumscribed to the western world only, but has also emerged as a challenge on a global scale and this has been proven in various similar researches in African contexts (37). Within India’s mental health framework for children, it has become imperative to integrate such insights so as to prepare the forthcoming generation by building up resilience to changes in the climate. Initiatives focusing on resilience which are school-based and placing of counselors of mental health in those schools which are struck by natural disasters can go a long way in helping the children in overcoming their trauma. When cyclones and floods happen the importance of mental health units which are mobile in nature can help in lowering the incidence of long-term sicknesses such as PTSD and offer psychological treatment (38–40). Climate change affects children’s psychological development through disrupted schooling, displacement, and eco-anxiety. While PTSD and depression are common post-disaster outcomes (33, 34), long-term impacts manifest in reduced concentration, academic decline, and heightened climate-related anxiety (31, 36). Thus, children’s vulnerability is distinct because of their developmental sensitivity and dependency on adult and institutional protection.

### The impact of climate change on women’s mental health in India

Climate change has a profound impact on women’s mental health in India, shaped by biological, socioeconomic, and cultural factors. As environmental conditions worsen, gender-based health inequalities are expected to increase (41). Though data is limited, studies show that women, particularly older or pregnant women, are more vulnerable to heat-related health issues (42, 43). The psychological state of women influences their mental health and this was found in a study by Parida (89). Social support and resilience also played a key role in this. Among the vulnerable section of people were those whose coping mechanisms were severely limited. Most of them had conditions that were present in them before and their experiences of life shapes their recovery psychologically. Research by Sorensen et al. (41) showed how an added layer of vulnerability is seen in cases of pregnancy. Those women often has to overcome various challenges due to changes in climate. It can also be considered how the physical and emotional turmoil arising out of their pregnancy are worsened by natural disasters. The support systems of those pregnant women can be further hampered by natural disasters such as heatwaves, droughts and floods. In such cases, their increased stress and anxiety further increases the risks of PTSD and hinders the development of the fetus and also the health of the mother. Their physiological differences such as reduced sweating, more subcutaneous fat, and higher metabolic rate makes them particularly weak in face of heat stress (41, 44).

Food security in India is severely hampered by changes in climate. Such shortages in food affects the mental health of women as their nutritional needs are increased due to menstruation, followed by pregnancy and consequent lactation (41). More frequent extreme weather events and disruptions in agriculture lead to food scarcity, worsened by cultural norms that prioritize food for children and men, which results in poor nutrition and rising anemia among women (45). Micronutrient deficiencies, common in food-insecure environments, are linked to cognitive issues such as reduced attention, impaired memory, emotional disturbances, and sensory perception problems (46). These cognitive and emotional challenges contribute to higher levels of stress, anxiety, and depression, impairing women’s ability to care for their families and engage in productive activities (41). In those areas which are vulnerable to natural disasters, regular screening of mental health and counseling must be incorporated into prenatal and postnatal care at maternal health clinics. Anxiety, risks of PTSD and depression can be minimized to a great extent by preparing the health workers of the community to provide supportive counseling to expecting women who have been displaced or affected by a disaster (47–49).

### The impact of climate change on the mental and physical health of older adults in India

The phenomenon of climate change represents a significant public health emergency, heightening the occurrence and intensity of natural disasters such as earthquakes, floods, and wildfires. These catastrophic events can have enduring psychological repercussions, particularly affecting older adults who are more susceptible to both physical and mental health challenges (50). Climate change leading to increased heat also increases the frequency of heat waves, worsening quality of air. Flooding and irregular rainfall are also similar effects of it, resulting in affecting the older adults and worsening their weakness to certain conditions such as tiredness due to heat, mostly which can cause strokes in them along with similar complications in their health (51). Their respiratory conditions are further hampered due to increase in air pollution. Their need to be hospitalized increases during times of severe natural disasters like wildfires and droughts because in those times the quality of air worsens to a great extent. The transmission of various diseases such as diarrhea and cholera are facilitated due to the rainy seasons which have become unpredictable. This is because such unpredictability compromises the hygiene and safety of drinking water. The fragile immune systems of the older adults are thus further put at risk by such conditions as their recovery periods are usually prolonged. Age-related vulnerabilities, encompassing anxiety, depression, post-traumatic stress disorder, and sleep disturbances, are increasingly magnified by the cascading effects of climate change, which destabilize both environmental and social determinants of mental health (50).

In India’s rural landscapes, where agricultural disruption translates into financial precarity, food insecurity, and inflated prices, older adults often reliant on fixed incomes experience compounded risks of malnutrition and declining physiological resilience, setting in motion a feedback loop of physical frailty and psychological distress (52). The erosion of familial and community support structures under these pressures forces many older adults to navigate illness and deprivation in isolation, a condition that research links directly to heightened rates of depression and cognitive decline in urban and semi-urban centers such as Kolkata (53, 54). Consequently, older adults are confronted with considerable mental health risks as a direct consequence of climate change (50, 51). Mental health geriatric units can be set up at primary health centers to address factors causing stress such as heat waves, social network loss, and relocation. Improvement of outreach programs which are home-based, helplines of the community, carer networks which volunteers for help might support the older adults survivors feel less alone and continue caring for them (55, 56).

### The impact of climate change on mental health in urban communities of India

The impact of climate change on mental health in urban India is increasingly evident, with cities facing challenges like high population density, pollution, extreme weather, and socio-economic inequalities. Urban areas are particularly affected by the Urban Heat Island Effect, where materials like concrete absorb and retain heat, leading to higher temperatures (57, 58). Heat waves are linked to a rise in mental health issues, including mood disorders, anxiety, dementia, and psychological fatigue, which can impair cognitive function, emotional stability, and productivity (29). There was a link between elevated workplace temperatures and heightened psychological distress (59). Consequently, residents in these urban environments frequently express feelings of being overwhelmed by the persistent threat of extreme heat, which exacerbates stress and contributes to the development of mood disorders. Another significant episode of urban flooding in India took place in Mumbai on July 26, 2005, when the city experienced an unprecedented rainfall of 994 mm within a single day, resulting in a complete halt of city activities (60). The subsequent flash floods and landslides led to the tragic loss of at least 425 lives, with an additional 216 fatalities linked to illnesses arising from the flooding (60, 61). The aftermath of the 2005 Mumbai flood also saw some individuals developing suicidal ideations as they faced overwhelming stress from the devastation of lives, homes, and property (61).

Urban laborers, especially those in the informal sector, face severe challenges from climate-induced disasters. For instance, construction workers, street vendors, and daily wage earners endured significant financial losses during the prolonged flooding that devastated the city. The resultant loss of income not only exacerbated economic instability but also contributed to increased levels of stress, a pervasive sense of despair, and a rise in mental health issues, including depression. The interconnection between air pollution and climate change is well established, with poor indoor air quality being associated with various neurological and psychological issues, including cognitive decline and behavioral changes (62). In November 2016, New Delhi faced one of its most severe air pollution crises, when the Air Quality Index (AQI) soared beyond 500, impacting the entire Indo-Gangetic Plain. The stagnant weather conditions deteriorated the situation, causing PM2.5 concentrations to rise dramatically from 142 μg/m^3^ to 563 μg/m^3^. Satellite observations revealed a 50–70% increase in pollution levels, primarily attributed to biomass burning in adjacent regions such as Punjab and Haryana (63, 64). Support services for psychological issues such as crisis hotlines, cooling centers with appointed counselors, and campaigns for awareness in communities that are not officially recognized, should be incorporated into city-level heat response plans. Services related to mental health need to get ready to handle eco-anxiety brought on by frequent flooding, pollution, and crises of climate in urban areas (65, 66).

### Impacts of climate change on mental health in rural areas of India

The effects of climate change on mental health in both urban and rural areas of India pose unique challenges, influenced by variations in environmental conditions, economic activities, infrastructure, and social interactions. Although both rural and urban environments encounter mental health challenges emanating from climate change, their manifestations diverge starkly, as rural regions whose economies depend heavily on agriculture and fishing experience profound psychological distress triggered by erratic weather patterns, droughts, floods, cyclones, and heatwaves, exemplified by the unprecedented May 2024 heatwave in Rajasthan, during which temperatures soared to 50 °C and prompted the India Meteorological Department to issue a red alert across several northern and western states, including Punjab, Haryana, Chandigarh, Delhi, western Uttar Pradesh, and Gujarat, warning against severe heat-related illnesses and heatstroke. The unprecedented heatwave that culminated in 122 fatalities 61 of which occurred between May 23 and May 30, 2024 set a new temperature record of 50.8 °C in Rajasthan’s Churu district, surpassing the 2019 benchmark and underscoring the intensifying trajectory of extreme thermal events in India’s climate landscape.

As escalating temperatures driven by anthropogenic climate change amplify heat exposure, agricultural laborers in rural India confront compounded vulnerabilities, where prolonged physical exertion under oppressive environmental conditions precipitates heat stress disorders that erode health, diminish productivity, and destabilize agrarian livelihoods (67). Research by Pailler and Tsaneva (68) highlights the negative impact of elevated temperatures on the psychological wellbeing of Indian adults, revealing that increased heat significantly exacerbates stress measured through sleep disturbances, coping abilities, and cognitive challenges along with a diminished sense of agency, particularly in rural populations compared to their urban counterparts. Interventions of psychological and economic nature which are linked are necessary for farmers. In those states plagued by drought, merging crop insurance with peer support groups, helplines of farmers, and psychiatric counseling has demonstrated promise in lowering distress and the risk of suicide (69, 70).

### Effect of climate change on mental health in coastal regions of India

India’s low-lying coastal zones, home to up to 310 million people, are highly vulnerable to climate change due to rising sea levels and frequent cyclones, especially along the eastern and southern coastlines. Coastal communities in states like Odisha, West Bengal, Kerala, and Tamil Nadu face threats from cyclones, tsunamis, flooding, and coastal erosion, disrupting economies and causing significant psychological distress. For instance, survivors of the 2004 tsunami in Tamil Nadu experienced severe psychological distress, with many showing symptoms of post-traumatic stress, particularly women, those with injuries, and those who lost children or financial security (71). Kar et al. (72) found high rates of psychiatric disorders like anxiety, depression, and PTSD among disaster survivors 4.5 years later, with key risk factors including direct exposure to the disaster, low education, and the loss of homes and livelihoods. Prolonged grief disorder (PGD) was common, especially among those who lost a spouse or had significant home damage. Disasters such as Cyclones Fani, Amphan, and Yaas have caused large-scale displacement, property loss, and livelihood destruction, and, as a study following Cyclone Fani in Odisha revealed high rates of PTSD, anxiety, depression, and elevated suicidal thoughts (73), the perpetual fear of such climate-related catastrophes continues to generate sustained worry and apprehension, compelling entire communities to relocate in search of safety.

Kar et al. (73) found strong link between mental illnesses and incidents such as property damage, evacuation, and displacement. He stresses the importance of enhancing disaster management for the solving of the psychological damage. The comparative analyses across various studies highlighted a clear gradient of impact where acute disasters (e.g., cyclones, floods) prominently trigger PTSD and acute anxiety, whereas chronic climate stressors (e.g., heatwaves, droughts) manifested as depression, hopelessness, and suicidality. This distinction underscores the necessity of differentiated policy responses tailored to the type and duration of climate exposure. Commonly, natural disasters such as cyclones and tsunamis cause a lot of disturbances in those areas where the primary source of income is fishing and fishing alone. In the wake of natural disasters the fishing population faces various loses and this in turn causes their families to be overwhelmed with immense grief and a looming sense of financial uncertainty. Such disruptions may also cause blocking of their access to local fishing grounds for months. This will significantly lower their income and contribute to financial hardship. This long-term hardship results in more depression and more anxiety. Economic losses such as those tend to act as mediating factors between climate stressors and psychological results where loss of livelihood, displacement, and social isolation contribute to symptoms of anxiety, depression, and trauma.

Disruption of support systems within communities and traditional means of living increases these mental health concerns, adding importance to the effective support of victims of climate catastrophes. In addition to families’ support groups and rehabilitation programs extending over greater lengths of time for dealing with bereavement, anxiety, and suffering because means of living, cyclone shelters and relief camps should have on-site counseling as well. Incorporating on-site counseling facilities within cyclone shelters and relief camps are essential and necessary along with mobile mental health units, which can help in early psychological intervention and thereby reduce long-term morbidity. Following numerous shocks that have disrupted the climate, communities in the coastal areas can recover their resilience through the assistance of sustained follow-up services (74–76).

### Climate change and mental health in agricultural communities

Heat and droughts usually shrink both yields and wages and they force a connection between financial despair and psychological breakdown (77). The weather’s unpredictable patterns with its droughts, its sudden floods rewrites livelihood and identity simultaneously. Each such storm becomes another action of disorientation (77). Water, whether withheld or left open, determines not only sustenance but sanity of the vulnerable sections of the population. Dependency somehow goes on to magnify fragility. Farmers remain linked to the whims of monsoon, to reservoirs and rivers, to snowmelt that sometimes never comes. A single season’s irregularity may cause empty granaries, unsettle the minds of the village folk, and drive away families. Floodwaters do more than drown fields, they cripple the whole system or order by which the affected areas function. Such a crippling result in schools being closed, homes drifted, debts deepened etc. (78). Across India’s agricultural belts, environmental degradation mixes with mental depletion, which in turn creates a crisis of ecology and emotion which are interconnected (79). Extreme weather no longer shocks but recurs. Farmer suicides during drought seasons haunt not just Australia’s outback but India’s heartland too gives us a similar picture (80). Failed crops lead to failed coping on the part of the farmers, and drought becomes both meteorological and psychological at the same time. Each unpredicted dry spell triggers anxiety, despair, and acts of self-destruction which causes debt repeating its cycle including harvest lost, loan renewed, hope deferred until poverty itself becomes a climate whereas prolonged exposure to heat brings not only resilience but also exhaustion, often ending fatally (29).

States like Karnataka, Maharashtra, Kerala, Andhra Pradesh, and Madhya Pradesh, all drought-prone, carry the highest suicide tolls (81). Maharashtra’s tragedy is sharply defined by its numbers. After the 2015 drought, the Marathwada region reported 4,700 suicides between 2015 and 2019 and 1,109 was reported in the first year itself (82). Data from Parida et al. (81) exposes drought as a primary catalyst: crop failure leads to debt, debt leads to despair, and despair very often leads only to death. Floods, on the other hand, do not carry the same statistical imprint. They only serve to add to the cumulative distress. Cotton-producing states like Karnataka, Maharashtra, and Kerala are some of the regions burdened by rural poverty. These states particularly highlight the densest clusters of farmer suicides, tracing the geography of tragedy directly to that of drought (81). Across populations, certain patterns emerge: acute disasters (cyclones, floods) predominantly trigger PTSD and anxiety, whereas chronic exposures (droughts, heatwaves) are linked to depression and suicide. However, resilience varies by social position women’s vulnerability is shaped by gendered caregiving roles; children’s by developmental stage and school disruption; and the older adults by isolation and declining physical health. Studies among rural communities emphasize livelihood loss, while urban populations report eco-anxiety and pollution-linked distress. Despite distinct contexts, all groups share exposure to inadequate post-disaster mental health support and policy neglect.

### Implications of climate change on mental health policies in India

The progressively building up of the weight of climate change places pressure not just on livelihood and land but also on the intricate layers of the human mind. It requires India to rethink its healthcare systems and national policies. The policies have, up until now, addressed psychological fallout as a peripheral issue instead of a central fault. The current mental health systems almost entirely miss the quiet undermining wrought by climate stressors, abandoning the most vulnerable sections. This includes the farmers, workers, and the displaced families all of whom are stranded in neglected suffering. Extreme weather events heat up and resources dwindle. Resilience, instead of simply being a slogan, must be embedded in the very design of healthcare. Climate change does not simply accompany mental illness, but it doubles it, and this is something that is usually ignored in most policies by most policymakers. It then connects poverty, inequality, and hopelessness into one tightening strand that tautens the very institutions that are supposed to heal and nurture. Workers in the rural areas along with urban underclass under withering heatwaves, also including coastal communities witnessing the sea come closer inch by inch each bear unique vulnerabilities that require climate-informed responses coupling immediacy with foresight. Disaster management strategies also needs to move beyond its logistical preoccupation with shelter and relief supplies. It needs to incorporate mental health interventions that can buffer trauma before it sets into hard and lasting suffering. The objective of it is that during the process of reconstruction of the homes, the country and its people do not fail to reconstruct its sanity too. There is an urgent need to reconstruct robust mental health infrastructure, enhance community-based interventions, and create public awareness to build resilience against climate adversity.

Moving forward practically is not merely a policy tweak but a reimagining of what we mean by “climate resilience.” India’s frameworks NAPCC, State Action Plans, NDMA directives often speaks volumes of crops, infrastructure, water, and energy, but rarely of the invisible wreckage left inside people’s minds. The irony is brutal: a village may receive new embankments against floods, but its people may drown quietly in nightmares that no blueprint can measure. Scholars have been contending for a long time that adaptation cannot be merely material; psychological aspects are inherent with climate survival (83, 84). Psychological first aid, trauma counseling, and ongoing mental health services ought not to come as benevolent afterthoughts after the cyclone has blown over. They need to walk alongside food rations and first aid kits, institutionalized in the very design of disaster preparedness. Picture a disaster response team where in addition to engineers and medics, a psychosocial counselor, trained and ready, is not an extravagance but a given presence someone who knows that survival is not just of the body but also of the mind, that resilience must be founded on unshattered psyches. This is not about creating new programs but stitching into existing cloth neglected strands.

The health-related missions are already established; the structures of disaster management are already in place. But where the lack lies is the willingness to accept that fear, sadness, and trauma are as tangible as broken bones. Climate adaptation without mental health is ultimately half a solution masquerading as whole (83, 84). Simply the integration of surveillance measures related to mental health into disaster responses cannot be considered the solution. On the contrary, it is the difference between noticing fractures when they are still minimal and waiting until they become prominent. Such a disaster-linked surveillance framework, when connected to platforms like the National Mental Health Survey, can successfully chart the seeming earthquakes of the mind as carefully as seismographs map the earth. Imagine community health workers equipped with mobile tools, logging symptoms in real time—not as passive record-keepers but as sentinels, catching distress before it hardens into despair. The effect would be twofold: individuals at risk could be swiftly guided toward care, and the system itself would learn to adapt with every entry, every crisis, every recovery. Such vigilance transforms disaster preparedness from reactive to anticipatory, reducing the hidden toll of psychological morbidity and turning resilience into something more than a slogan (28, 85).

### Psychological and community-based interventions

#### Individual-level psychological interventions

Evidence-based psychological interventions play a critical role in mitigating climate-related mental health impacts. Cognitive-behavioral therapy (CBT), trauma-focused therapy, and mindfulness-based stress reduction have demonstrated efficacy in reducing anxiety, depression, and PTSD symptoms following environmental disasters (40, 90). Interventions should be adapted for cultural relevance and delivered through both in-person and digital formats, especially in disaster-prone and rural regions where access to services is limited (38). Incorporating stress management, relaxation training, and psychoeducation within primary care can help individuals develop adaptive coping strategies and strengthen psychological resilience.

#### Community-based and culturally grounded strategies

Community-level interventions are essential for fostering collective resilience and recovery. Peer-support groups, self-help collectives, and village mental health committees can facilitate mutual aid and early identification of distress (55, 56). Evidence from Kerala’s *Kudumbashree* women’s network demonstrates how social cohesion enhances post-disaster recovery (91). Integrating indigenous practices, such as faith-based rituals and traditional healing, can enhance community acceptance and sustainability (79). Evaluations of post-cyclone community outreach programs in Odisha and Tamil Nadu have shown reductions in PTSD and depressive symptoms over 12 months (73), underscoring the value of culturally tailored psychosocial responses.

#### Professional training and capacity building

Building the skills of health and social service professionals is crucial for effective disaster mental health response. Training programs for ASHA workers, primary care physicians, and psychiatric social workers should include modules on trauma-informed care, disaster psychology, and culturally sensitive counseling. Short-term certificate courses and continuing education in climate-mental health linkages can strengthen frontline preparedness (86, 87). Integrating psychosocial support protocols into the curricula of medical, nursing, and social work programs ensures that future professionals are equipped to respond rapidly and effectively during environmental crises.

### Implications for social work practice and research gaps

Social workers are uniquely positioned to operationalize climate-informed mental health strategies at both micro and macro levels. Their roles encompass psychosocial assessment, advocacy for climate justice, and community mobilization during and after disasters (92). Integrating environmental social work principles into practice enables professionals to address the ecological determinants of mental health. Future research should focus on (a) evaluating the effectiveness of community-based psychosocial programs, (b) conducting longitudinal studies on resilience trajectories, (c) developing culturally validated tools for assessing climate-related distress, and (d) implementing digital mental health models in remote settings.

## Strengths of the review

The strength of this narrative review lies in its comprehensive and integrative examination of the intersection between climate change and mental health within the Indian context, encompassing diverse vulnerable populations such as children, women, older adults, and rural and coastal communities. By synthesizing multidisciplinary evidence from psychology, social work, public health, and environmental sciences, the review advances a holistic understanding of how climatic stressors interact with social and economic determinants to shape mental health outcomes. The inclusion of both national and international literature enhances contextual depth, while the use of an eco-social and climate vulnerability framework provides conceptual coherence across sections. Furthermore, the review highlights critical policy gaps, culturally grounded intervention models, and the emerging role of social work in climate resilience, thereby contributing valuable insights for research, clinical practice, and evidence-based policymaking in the field of climate–mental health linkages in India.

## Limitations of the review

The present narrative review has several limitations that warrant consideration. Methodologically, as a narrative rather than a systematic review, it may be subject to selection bias and lacks quantitative synthesis, which limits the ability to derive pooled estimates or causal inferences. The inclusion of heterogeneous sources ranging from peer-reviewed studies to policy documents introduces variability in methodological rigor, while the absence of a standardized quality appraisal framework may affect the reliability of certain findings. In terms of the underlying evidence base, the available research from India is largely cross-sectional, geographically uneven, and dependent on self-reported data, constraining generalizability and precision. Moreover, community-based intervention studies are fragmented, often pilot in nature, and lack longitudinal outcome evaluation. These factors collectively restrict the interpretive depth and policy applicability of the findings, underscoring the need for future research that is longitudinal, regionally representative, methodologically standardized, and integrated across health, environmental, and social sectors to strengthen the evidence base for climate–mental health policy and practice in India.

## Conclusion

The present paper examines the link between climate change and mental health in vulnerable populations in India, highlighting its widespread effects on various groups. As the country experiences rapid economic growth alongside climate change, addressing the mental health impacts of environmental challenges becomes more urgent. The research emphasizes how climate change worsens mental health issues, especially for women, the older adults, children, and rural populations. Extreme weather events like heatwaves, floods, cyclones, and droughts are major stressors. For women, particularly pregnant women, climate-related disasters intensify anxiety, depression, and PTSD. The older adults, facing unique health challenges, are more vulnerable due to extreme weather, health risks, and disrupted support networks. Rural communities dependent on agriculture are hit hard by erratic weather and crop failures, leading to greater economic and psychological strain. Urban areas are also affected, with the Urban Heat Island Effect worsening mental health problems. Events like the Wayanad landslides highlight the severe mental health impacts of climate change, calling for urgent action. The study advocates for a comprehensive mental health strategy that incorporates climate change, urging policymakers and mental health practitioners to consider climate factors in their plans. Prioritizing mental health support, building community resilience, and integrating climate considerations into mental health policies are essential steps to mitigate the effects on vulnerable populations. Proactive measures are needed to protect the mental wellbeing of affected communities.
